# Factors That Predict the Progression of Non-alcoholic Fatty Liver Disease (NAFLD)

**DOI:** 10.7759/cureus.20776

**Published:** 2021-12-28

**Authors:** Madhangi Parameswaran, Hamzah A Hasan, Jafor Sadeque, Sharan Jhaveri, Chaithanya Avanthika, Abimbola E Arisoyin, Maulik B Dhanani, Swaroopa M Rath

**Affiliations:** 1 Radiology, Massachusetts General Hospital, Boston, USA; 2 School of Medicine, Hashemite University, Zarqa, JOR; 3 Internal Medicine, Al Mostaqbal Hospital, Jeddah, SAU; 4 Internal Medicine, Smt. Nathiba Hargovandas Lakhmichand Municipal Medical College, Ahmedabad, IND; 5 Medicine and Surgery, Karnataka Institute of Medical Sciences, Hubli, IND; 6 Internal Medicine, College of Medicine, University of Lagos, Lagos, NGA; 7 Internal Medicine, Southwestern University School of Medicine, Cebu City, PHL; 8 Medicine, Srirama Chandra Bhanja Medical College and Hospital, Cuttack, IND

**Keywords:** treatment choices, mitochondrial senescence, demographics, long-term prognosis, disease progression, human genetics and epigenetics, nonalcoholic fatty liver disease (nafld)

## Abstract

Non-alcoholic fatty liver disease (NAFLD) refers to a spectrum of diseases involving the deposition of fat in the hepatocytes of people with little to no alcohol consumption. NAFLD is associated with hypertension, diabetes, obesity, etc. As their prevalence increases, the propensity and severity of NAFLD might increase. As per the recently developed multi-hit hypothesis, factors like oxidative stress, genetic predisposition, lipotoxicity, and insulin resistance have been found to play a key role in the development of NAFLD and its associated complications. This article focuses on NAFLD, its pathophysiology, risk factors, and the various genetic and epigenetic factors involved in its development along with possible treatment modalities.

We conducted an all-language literature search on Medline, Cochrane, Embase, and Google Scholar until October 2021. The following search strings and Medical Subject Heading (MeSH) terms were used: “NAFLD,” “NASH,” “Fibrosis,” and “Insulin Resistance.” We explored the literature on NAFLD for its epidemiology, pathophysiology, the role of various genes, and how they influence the disease and associated complications about the disease and its hepatic and extrahepatic complications. With its rapidly increasing prevalence rates across the world and serious complications like NASH and hepatocellular carcinoma, NAFLD is becoming a major public health issue and more research is needed to formulate better screening tools and treatment protocols.

## Introduction and background

Non-alcoholic fatty liver disease (NAFLD) refers to a spectrum of diseases caused by deposition of fats in more than 5% of hepatocytes, primarily in the form of triglycerides in the absence of alcohol consumption (over 21 drinks/week for men and 14 drinks/week for women), use of medications causing hepatic steatosis, or hereditary and autoimmune causes [1]. The histological presentation can range from the bland accumulation of triglycerides within the hepatocytes (fatty liver) to non-alcoholic steatohepatitis (NASH). Fatty liver is a benign non-progressive form of the disease, whereas NASH is associated with inflammation, hepatocyte ballooning, and deposition of collagen fibers that can eventually progress to cirrhosis and liver cancer [2].

After Mayo Clinic first described the occurrence of NAFLD in 1980, the pathogenesis has been widely studied, although the exact mechanism is still unknown [3,4]. According to previous research that describes the “two-hit hypothesis,” the first strike is represented by increased hepatic triglyceride content and resistance to insulin. This vulnerable liver is then subjected to a second hit, which comprises inflammatory cytokines, adipokines, mitochondrial dysfunction, and oxidative stress [5]. At present, the “multiple hit model” is the commonly accepted idea that involves the association with metabolic dysfunction and interactions of complex environmental and genetic influences. NAFLD is now considered the hepatic manifestation of metabolic syndrome and is closely associated with high triglyceride level content, low high-density lipoprotein (HDL), hypertension, and morbid obesity [6].

As it requires histological diagnosis, the gold standard for diagnosis of NAFLD is liver biopsy. But specific non-invasive modalities are acceptable, including hepatic ultrasonography (USG), abdominal computed tomography (CT) scan, and magnetic resonance imaging (MRI) [7]. Serum biomarkers such as alanine aminotransferase (ALT) and aspartate aminotransferase (AST) have been used for screening [8]. The prevalence of NAFLD in the general population in various nations is estimated to range from 3% to more than 24% using blood markers [8]. NASH affects around 6-8% of individuals in the United States (about 25% of those with NAFLD) [9]. If these studies are assumed to represent the wider population, it would imply cirrhosis in 1.5-2% of the US population owing to NAFLD [9].

While the progression to cirrhosis is rare, subsequent chances of development to primary liver cancer can be as high as 3%. The incidence of hepatocellular carcinoma and intrahepatic cholangiocarcinoma associated with NAFLD is gradually increasing [10]. Documentation of progressive hepatic fibrosis is used as an important surrogate marker for advanced liver disease. In recent studies, the annual fibrosis progression rate was found to be 0.09 times (95% CI: 0.06-0.12) in patients with baseline NAFLD [11]. In the United States, NASH is the second most common cause of liver disease among transplant recipients [12]. With the rise in the incidence of obesity, hypercaloric diet, and sedentary lifestyle, NAFLD has become the dominant form of chronic liver disease among the pediatric and adolescent age group, with cases detected as young as three years [13,14]. Over the last decade, many treatments have been developed and tested, but no effective treatment is currently available [15]. Thus, it becomes crucial to identify the demography at the highest risk and the elements that influence the advancement of NAFLD.

## Review

Pathogenesis and molecular mechanisms of NAFLD

Typically, two pathologically different conditions are identified: non-alcoholic fatty liver (NAFL) and NASH. On the one hand, NAFL is characterized by simple liver steatosis; on the other hand, NASH is identified by the presence of macrovesicular fat accumulation, hepatic ballooning, and lobular inflammation with or without any fibrosis [16-18].

NAFLD is a multifactorial disease, and its pathogenesis and progression are not fully understood. It is a complex disease that interacts with other organs in the body, for instance, on the intestine-liver-adipose tissue axis for its development. Multiple pathways have been proposed over the past years on how NAFLD progresses [19]. Although the most famous theory to explain NAFLD is the “two-hit hypothesis,” a more accurate explanation of NAFLD pathogenesis explained that several molecular and metabolic changes occur together in its development and progression. This “multiple hit” hypothesis considers multiple insults acting in concert to cause NAFLD [19,20].

Lipotoxicity and NAFLD

Hepatic lipotoxicity happens when the liver's capacity to use, reserve, and export free fatty acids (FFAs) as triglycerides (TGs) is saturated by a massive FFA influx from the periphery, mainly the adipose tissue or by increased hepatic de novo lipogenesis [21-23].

FFAs are exported from the liver as very-low-density lipoprotein (VLDL) or stored in hepatic lipid droplets after being metabolized via mitochondrial β-oxidation or re-esterification into TGs inside the hepatocytes. Lipid droplets inside the hepatocytes undergo lipolysis and release fatty acids into the hepatocyte FFA pool [24-26]. Lipotoxic species formed from excess FFA inside liver cells cause endoplasmic reticulum inflammation, oxidative stress, and activation of inflammatory mediators [27-29].

Insulin Resistance and NAFLD

Insulin resistance plays a vital role in the pathogenesis of NAFLD [30]. The role of insulin in our body is to stimulate glucose utilization and increase lipid accumulation in insulin-sensitive organs such as skeletal muscle, adipose tissue, and liver. Insulin resistance leads to increased lipolysis in dysfunctional adipose tissue and reduced glucose uptake in skeletal muscle [27,28]. As a result, there is an increased FFA in the bloodstream and the liver as well. Higher hepatic uptake of FFA leads to the accumulation of TGs in the liver and the release of VLDL via fatty acid esterification.

In contrast, glucose uptake contributes to a lesser extent via de novo lipogenesis, leading to steatosis (NAFL) and dyslipidemia [26]. Studies have shown metabolic cross-talk between liver and adipose tissue [30]. Some peptides released by adipose tissue, such as adiponectin and interleukin 6 (IL6), have both proinflammatory and protective effects on the liver [31,32]. The enzyme dipeptidyl peptidase 4 (DPP-4) can promote insulin resistance. This enzyme, secreted by hepatocytes, has been seen to work on plasma factor Xa to stimulate macrophages within the visceral tissue of mice. This mechanism is known to promote insulin resistance, emphasizing the important cross-talk between the liver and other tissues underlying metabolic dysregulation in NAFLD [33].

Oxidative Stress

Oxidative stress plays an important role in the pathogenesis of NAFLD [34,35]. Oxidative stress means the imbalance between the production of reactive oxygen species (ROS) and the scavenging capacity of the antioxidant system [36]. Mitochondria are the most important source of ROS in NAFLD, mainly due to increased fatty acid oxidation (FAO) [37]. At high concentrations, ROS cause oxidative modifications to cellular macromolecules (DNA, lipids, proteins, etc.) and cause accumulation of damaged macromolecules, initiating liver injury [35].

ROS can initiate lipid peroxidation by targeting polyunsaturated fatty acids, resulting in highly reactive aldehyde products such as 4-hydroxy-2-nonenal (4-HNE) and malondialdehyde (MDA). These reactive compounds have longer half-lives than free radicals and can diffuse into the extracellular space to boost tissue damage. Multiple studies have reported that both MDA and 4-HNE are increased in experimental animal models of NASH and in NASH patients compared to patients with NAFL [35-38].

Cytochrome P450, lipoxygenase, and cyclooxygenase, well-characterized pro-oxidant systems, combined with free-radical products, have been involved in the early stages of NAFLD [39,40]. On the contrary, a reduction in the activity of antioxidant enzymes such as catalase, glutathione peroxidase, glutathione S-transferase, superoxide dismutase, as wells as ROS scavengers (ascorbic acid, glutathione, α-tocopherol, ubiquinone, thioredoxin, and bilirubin) is a characteristic of livers from NASH patients [38,39,41].

Immune Cells Involved in NAFLD

Multiple immune cells are involved in developing NAFLD, such as activated Kupffer cells, the liver's resident macrophages, in combination with the recruitment of proinflammatory, monocyte-derived macrophages and neutrophil leukocytes from circulation [42,43]. With the substantial accumulation of macrophages, their polarization appears “proinflammatory” at the expense of anti-inflammatory and repair macrophages, likely because of the high abundance of proinflammatory cytokines such as interferon-γ, tumor necrosis factor (TNF), and lipopolysaccharide in NASH [44].

Epidemiology and risk factors of NAFLD

NAFLD has been increasing remarkably worldwide in recent years. It is estimated that the current prevalence of NAFLD exceeds 24% of the population globally [11], which implicitly indicates the increase in liver-related morbidity and mortality in the next few years [45,46]. The main cause for concern of NAFLD (particularly NASH) is that it is one of the commonest causes of hepatocellular cancer (HCC) and is expected to supersede viral hepatitis as the most common etiology for HCC in the United States due to the rapidly growing epidemic of metabolic syndrome [47,48]. NASH is also considered an increasing cause of liver transplantation in the US, UK, and also in developing countries [49].

Although the NAFLD disease spectrum ends with cirrhosis, hepatocellular carcinoma (HCC), cardiac events, and chronic kidney disease (CKD) are the major causes of death among NAFLD patients [50]. In addition, one study suggested a relationship between multiple NAFLD comorbidities and the poor prognosis of coronavirus disease 2019 (COVID-19) in NAFLD-infected individuals [51]. Due to its wide-ranging effects on multiple body systems, it is important to initiate a non-invasive screening method to detect NAFLD early.

Tables 1, 2 show the estimated prevalence of NAFLD globally according to regions and race, respectively. The highest prevalence is in the Middle East and South America (around 30%) and the lowest in Africa (13%) [11]. The effect of gender on NAFLD progression is not clear [52], but recent studies showed that males are more likely to develop NASH [53,54].

**Table 1:** Prevalence of NAFLD in different regions. NAFLD, non-alcoholic fatty liver disease.

Region	Estimated prevalence	Notes
North America	27%-34% [55]	N/A
South America	31% [11]	Brazil reported the highest NAFLD prevalence and Peru the lowest [56]
Europe	25% [11]	Varies by countries with a reported low prevalence rate of 8% in Romania to a reportedly high prevalence of 45% in Greece [57]
Asia	15%-20% [58]	These rates are higher in urban areas than those reported from rural areas indicating the highly increasing change of Asian lifestyle in urban areas [11]
Africa	13% [11]	Could be low due to a poor reporting system [59]
Middle East	32% [11]	N/A

**Table 2:** Prevalence of NAFLD in different ethnicities. NAFLD, non-alcoholic fatty liver disease.

Race	Estimated prevalence	Notes
Hispanic Americans	45% [60]	Highest prevalence
African Americans	24% [60]	Lowest prevalence
European American	33% [60]	N/A
Asians American	18% [61]	N/A
Hispanics Mexicans	33%	Higher prevalence of NAFLD than Hispanics of Dominican origin (16%), and Hispanics of Puerto Rican origin (18%)

Risk Factors of NAFLD

There are many established risk factors for NAFLD development and progression. A strong association has been found between diabetes mellitus type 2 (DM2) and NAFLD as a large proportion of DM2 patients have NAFLD. It is thought that insulin resistance plays a critical role at the cellular level in the pathogenesis of both conditions [62]. Other risk factors are somehow related to DM2, as obese people, who are at higher risk of DM2 [63], have a higher prevalence of NAFLD.

A study showed that >95% of patients with morbid obesity undergoing bariatric surgery would develop NAFLD in their life [64]. Because of this strong relationship between obesity and NAFLD, waist circumference has been suggested as a simple indicator for the risk of NAFLD development [65]. In addition, weight reduction strategies are emphasized as a preventive method of NAFLD development and progression [66]. A sedentary lifestyle and poor eating habits (high fructose (sugar) drinks and fatty food) are shared risk factors for the development of NAFLD and DM2 [67,68].

Studies among the Asian population showed that sarcopenia, the loss of skeletal muscle mass, has also been associated with the increasing severity of NAFLD [69]. This association needs further validation in the western world [69,70]. Women with subclinical metabolic disturbances are at higher risk of developing NAFLD after menopause, highlighting the protective role of estrogen in women before menopause [71].

Some studies stated that NAFLD could pass through generations, for instance, the metabolic phenotype of obese pregnant women could be transmitted to offspring, making them more susceptible to obesity and NAFLD [53,54]. Regarding gender, it is widely accepted that the male sex has an increased incidence of NAFLD. However, the role of sex in NAFLD progression remains inconclusive. Some studies report sex as an independent predictive factor of NAFLD severity, while others suggest exposure to estrogen to be a better metric [72]. The effect of sex hormones needs to be investigated further to elucidate their role.

Factors for NASH Development

Most studies are in agreement with shared factors that lead to NASH development. Factors associated with NASH development are body mass index (BMI) > 27 and lower hip-to-waist ratios. In addition, specific laboratory investigations were statistically significant, as elevated levels of ALT, AST, and serum sphingolipids (particularly ceramide) with low levels of HDL cholesterol were all associated with NASH development and progression [73-77]. Recently, a study by Tasneem et al. came up with a scoring system called the GULAB (based on gender, US liver findings, lipid (fasting) levels, ALT level, and BMI) score. It consists of several non-invasive parameters with a cutoff point of 5 associated with NASH development [74]. Another study by Seko et al. considered persistent elevation of ALT despite treatment as a valid indicator of NASH histological progression. It recommended strict control of serum ALT to prevent NAFLD progression [77].

Factors Inducing Advanced Fibrosis in NAFLD Patients

NASH patients with advanced fibrosis have a higher risk of developing liver cirrhosis that ends with liver cell failure [78]. Established factors associated with advanced fibrosis in NAFLD patients were male gender, age > 40 years, Caucasian ethnicity, DM2, hypertension, and a lower hip-to-waist ratio. Also, AST/ALT ratio > 1 and raised gamma-glutamyl transferase (GGT) were also noted in advanced fibrotic liver patients [73,74,79]. Thus patients with NASH are advised to check for cirrhosis in case of the presence of these parameters. Patients with NASH are at high risk of developing fibrosis, while patients with NAFL were a point of conflict, as many studies considered their condition as benign that would not develop liver fibrosis [80,81]. On the other hand, more recent studies found that NAFL patients may end up with liver fibrosis, but the progression is slower than NASH patients [79,82].

Genetic and epigenetic factors

Family studies and twin studies estimate the heritability of NAFLD between 20% and 70%, suggesting a vital genetic component for its development and progression [83]. The evolution of genome-wide association studies (GWAS) and candidate gene studies have helped identify numerous genes involved in lipid handling, insulin signaling, and even innate immunity. A select number of genes are summarized in the table below (Table 3). The most robust evidence across multiple ethnicities is for the patatin-like phospholipase domain-containing protein 3 (PNPLA3) gene, substituting isoleucine with methionine [84,85]. It produces a dysfunctional lipase enzyme that interferes with triglyceride metabolism and increases the probability of hepatic steatosis [86]. In addition, the PNPLA3 variant is also associated with the development of fibrosis and a five-fold increase in the risk of HCC [87]. The membrane-bound O-acyl transferase 7 (MBOAT7) gene involved in phospholipid metabolism has also been identified to increase the risk of NAFLD development and progression, particularly in the European population [88-90]. This gene has also been associated with a two times increase in risk for HCC [91]. The transmembrane 6 superfamily member 2 (TM6SF2) gene has also been implicated in NAFLD incidence and hepatic fibrosis development in Europeans [92,93]. This gene is involved in liver secretion of very-low-density lipoprotein; a loss-of-function mutation increases hepatic steatosis while protecting cardiovascular disease (CVD) risk [94]. However, this association has not been replicated in other ethnicities [95]. Table 3 is a brief summary of the various genes associated with NAFLD development and progression.

**Table 3:** Genes associated with NAFLD progression with a short description of their biochemical processes. NAFLD, non-alcoholic fatty liver disease; HCC, hepatocellular carcinoma; VLDL, very-low-density lipoprotein; PNPLA3, patatin-like phospholipase domain-containing protein 3; MBOAT7, membrane-bound O-acyl transferase 7; TM6SF2, transmembrane 6 superfamily member 2.

Gene	Variant	Biochemical effects	Effect on NAFLD
PNPLA3	rs738409	Triglyceride metabolism	Increases incidence, progression, and HCC risk [84-87]
MBOAT7	rs641738	Phospholipid metabolism	Increases incidence, progression, and HCC risk [88-91]
TM6SF2	rs58542926	VLDL secretion	Increases incidence and progression [92-93]
Glucokinase regulator (GCKR)	rs780094	De novo lipogenesis	Increases incidence and progression [96]
17β-hydroxysteroid dehydrogenase 13 (HSD17B13)	rs72613567	Possibly lipid-related inflammation	Decreases progression [97]
Interleukin - 28B (IL28B)	rs12979860	Innate immunity	Decreases fibrosis [98]
Superoxide dismutase 2 (SOD2)	rs4880	Oxidative stress protection	Increases fibrosis [99]

Genetic risk scores are proposed to stratify the severity of NAFLD based on the identified variants [100]. A recent study has demonstrated a better prediction of HCC risk when using genetic risk scores combining the PNPLA3, TM6SF2, glucokinase regulator (GCKR), MBOAT7, and 17β-hydroxysteroid dehydrogenase 13 (HSD17B13) variants [101].

Recent GWAS studies have identified new candidate genes, which may contribute to the genetic risk of NAFLD. For example, associations between a variant in the sorting and assembly machinery component 50 (SAMM50) and NAFLD incidence are reported in Asian and Mexican populations [102]. In addition, variants in glycerol-3-phosphate acyltransferase, mitochondrial (GPAM) and apolipoprotein E (APOE) genes are also identified to be associated with liver fat in the European population [103]. Validation studies need to be performed to understand the effects of the identified variants, which may add to the development of more robust genetic risk scores.

Epigenetics

Epigenetics is described as genetic modification without changes to deoxyribonucleic acid (DNA) sequences to cause a phenotypic change. Its role in the progression of NAFLD via DNA methylation, histone modification, and changes in microRNA (miRNA) expression is gathering increasing attention [94]. DNA methylation refers to the transfer of a methyl group to form 5-methylcytosine at cytosine-guanine dinucleotide (CpG) islands to reduce gene transcription [104].

Changes in methylation of CpG islands in the PNPLA3 gene and fibrogenic genes such as platelet-derived growth factor (PDGF) and transforming growth factor beta (TGFβ) have been associated with the progression of fibrosis [105]. A recent study has demonstrated a change in expression of 288 genes in samples with fibrosis along with a change in cell composition driven by DNA methylation [106]. This highlights the potential of DNA methylation to be the driving factor of fibrosis in NAFLD. Methylation of mitochondrial genes such as NADH dehydrogenase 6 (MT-ND6) in the liver has also been associated with more severe forms of NAFLD [107].

Recent studies have shown a dietary component in DNA methylation as methylation is driven by folate-dependent methyltransferases [104]. It has also been demonstrated that a high-fat diet leads to hypermethylation of peroxisome proliferator-activated receptor gamma (PPARγ), which is linked to triglyceride accumulation [108]. Hence, dietary interventions could act as a potential therapy for the reversal of NASH.

Changes in acetylation of amino acid residues of histone tails driven by histone acetylases (HATs) and histone deacetylases (HDACs) have been shown to affect inflammatory responses, which drive the development of NAFLD [84]. For example, increased activity of HAT has been described to increase transcription of hepatocyte carbohydrate-responsive element-binding protein (chEBP), a well-described activator of genes involved in acute phase response and lipogenesis [109]. Sirtuins (SIRT), a type of HDAC, attenuates responses to metabolic stress [110]. SIRT1 is demonstrated to have a protective effect on NAFLD and other metabolic conditions and its deletion was associated with increasing severity of hepatic steatosis [110,111]. Lately, a murine knock-out model has shown that a lack of SIRT2 is associated with increased lipogenesis and fibrosis, suggesting a crucial role for histone modification in the NAFLD progression to fibrosis [112].

miRNAs are non-coding RNAs that target messenger RNAs for degradation and regulate their expression [94]. Modifications in the hepatic miRNA profile have been associated with NASH and HCC [113]. Mi-122, the most common miRNA in the liver, has been associated with tumorigenesis due to the deletion of its tumor suppressor effects [113]. It is also associated with NASH and liver fibrosis by activating tissue remodeling genes [94]. Upregulation of mi-192 is also associated with an increased incidence of NASH and has been proposed to be part of the miRNA panel to identify NASH [114]. In addition, mi-166 has shown an association with fibrosis in the Chinese population [115]. Other miRNAs such as mi-21, mi-34a, and mi-16 have also been associated with NASH [94]. Recently, miR-374a, miR-143, miR-1, miR-23a, and miR-423 have also been associated with NASH and target mitochondrial genes and genes involved in lipogenesis, apoptosis, and fibrogenesis [116]. Further research is necessary to validate these miRNAs to develop non-invasive biomarkers.

Mitochondrial senescence

Whatever the primary etiology of NASH, mitochondrial senescence (especially respiratory chain insufficiency) plays a major role in the physiopathology of the disease. Senescence is a form of cellular adaptation to various normal and abnormal cellular processes. It follows different forms of stresses such as changes in the structure of chromatin, stress involving oxidation, and activation of oncogenes [117]. Senescence in hepatocytes and other supporting cells of the liver such as sinusoidal endothelial cells, hepatic stellate cells, and Kupffer cells are closely related to the development and progression of NAFLD [118].

Mitochondrial changes in senescent cells include increased size and amount of tricarboxylic acid byproducts, an increase in the number of mitochondria, ROS, and proton leaks, and a decrease in the function and the membrane potential [119,120]. In 2001, a study conducted by Sanyal et al. found many morphological differences in the mitochondria of a normal liver and that of a NASH patient. NASH was linked with swollen and round mitochondria, clear matrix, loss of mitochondrial cristae, and multiple paracrystalline inclusions, although no indication of a widespread deficiency in fatty acid beta-oxidation was found in any of the groups [121]. Intramitochondrial paracrystalline bodies were associated with NASH with very strong specificity compared with normal controls, fatty liver, and hepatitis C patients.

Though the beta-oxidation of fatty acids was not much affected in the hepatocytes, the defective respiratory chain in the hepatocyte mitochondria generated increased ROS. In a lipid-rich microenvironment, ROS production increases lipid peroxidation, which is toxic to the hepatocytes. ROS harms the respiratory chain due to oxidative damage to the mitochondrial DNA, which starts a vicious cycle [122]. The involvement of mitochondrial senescence in the pathophysiology of NAFLD was reinforced when studies concluded the association between drugs causing steatohepatitis and mitochondrial dysfunction. Drugs like amiodarone or perhexiline cause impairment of electron transfer in the respiratory chain and inhibit beta-oxidation leading to dysfunctional hepatocyte and death [123].

When senescence occurs, its degree is directly proportional to the extent of NAFLD. Some markers support senescence in the liver with NAFLD, and this includes p53, p21, p16, and, more specifically, senescence marker protein-30 (SMP30) [124-126]. SMP30 is involved in ROS and glucose breakdown and maintains calcium balance, but levels of SMP30 reduce with age [127]. Studies have shown that SMP30 reduces further in NAFLD [127]. A decrease in the amount of SMP30 causes fat accumulation in the liver and eventual death of hepatocytes due to the disruption of its metabolism [128].

13C-ketoisocaproic acid (KICA) and methionine are markers of mitochondrial function. Breath tests using them as biomarkers showed a significant reduction in carboxylation in patients of NASH but not in patients with fatty liver [129]. Due to high localization in the mitochondria, the urea cycle metabolites have also been used as a non-invasive biomarker for staging and prognosis of NAFLD [130]. These novel methods should be used in the early detection and limitation of progression of NAFLD. However, it remains unclear whether the link between mitochondrial defects and NASH is the cause or an effect of the disease.

Complications and prognosis of NAFLD

Hepatic Complications

As previously mentioned, NAFLD is a spectrum of diseases mainly composed of patients with simple steatosis. In some patients, it can progress to a more advanced disease characterized by NASH and liver fibrosis and cirrhosis, or even HCC [131]. In a retrospective analysis of 619 patients with NAFLD, Angulo et al. concluded that the severity of liver fibrosis is associated with increased long-term mortality and other liver-related complications [132]. However, a collaborative cohorts study by Bhala et al. showed that patients with NAFLD (biopsy-confirmed) with advanced fibrosis had lower rates of hepatic complications and HCC in comparison with patients with hepatitis C virus (HCV) infection, but overall mortality was the same in both groups [133].

The prevalence of HCC among NAFLD patients is increasing, particularly in developed countries, as suggested by Kim and El-Serag [134]. Currently, limited studies have suggested exact mechanisms of HCC in NAFLD patients. On the other hand, in a cohort study consisting of 130 NAFLD patients, Kanwal et al. concluded that there was an increased risk of HCC and cirrhosis with stepwise addition of each metabolic trait. Their results showed that type 2 diabetes mellitus (T2DM) conferred the highest risk of progression to HCC and suggested T2DM is an essential target for secondary prevention [135].

As with the increasing incidence of NASH, the incidence of NASH-related liver transplantation is increasing. A retrospective cohort study was done by Cholankeril et al. concluding that NASH is the most rapidly growing indication and currently the second leading indication for liver transplant [136].

Extrahepatic Complications

NAFLD is a multisystem disease and is closely associated with many extra-hepatic complications such as CVD, CKD, and certain malignancies. Among all, CVD is the most common cause of death in patients with NAFLD [137,138]. A meta-analysis including 16 observational prospective and retrospective studies by Targher et al. showed that patients with NAFLD had a higher incidence of CVD events than patients without NAFLD [139]. In another meta-analysis involving 27 cross-section studies, Oni et al. concluded that there is a strong association of NAFLD with several CVD complications such as increased carotid wall thickness, impaired flow-mediated vasodilation, increased arterial stiffness, and subclinical atherosclerosis, which is independent of traditional CVD risk factors and metabolic syndromes [140].

The link between NAFLD and the development of T2DM has been well established by several studies in the past decade; however, the mechanism linking both conditions is multifactorial [141].

As previously mentioned, CKD is a well-established complication of NAFLD. Patients with NAFLD/NASH have a higher prevalence of CKD than patients without NAFLD/NASH. Although the exact pathogenic mechanism linking NAFLD and CKD is not well understood, it is believed that proinflammatory mediators released as a result of liver inflammation in patients with NAFLD cause an increase in pro-oxidative and pro-fibrotic molecules, which play a key role in the mechanism of CKD [142].

Several studies conducted in recent years have established an important link between NAFLD and colorectal carcinoma (CRC) and suggested NAFLD as an independent risk factor for the development of colon polyps and eventually colorectal adenocarcinoma. Inflammation of the liver due to NAFLD causes increased levels of proinflammatory and procoagulant factors and decreases levels of adipokines and adiponectin (protective factors), thus contributing to the pathogenesis of CRC in patients with NAFLD [143]. A correlation between NAFLD and psoriasis with similar pathogenesis (increased proinflammatory cytokines and reduced anti-inflammatory cytokines) has also been demonstrated [144].

A cross-sectional study by Gutierrez-Grobe et al. at the university hospital in Mexico City involving 197 women showed a significant association between NAFLD and polycystic ovarian syndrome (PCOS) and concluded that the prevalence of NAFLD was higher in postmenopausal women and women with PCOS than premenopausal women [145]. In the past few years, several experiments have suggested a possible connection between chronic intermittent hypoxia of obstructive sleep apnea syndrome (OSAS) and NAFLD. A meta-analysis done by Musso et al., including 18 cross-sectional studies, established a link between OSAS and a higher risk of NAFLD [146].

Treatment of NAFLD

Weight loss through lifestyle modifications such as hypocaloric diets and physical exercise remains the primary and most effective treatment for NAFLD of all stages [147]. The American Association for the Study of Liver Disease (AASLD) recommends a minimum of 3-5% loss of body weight to improve hepatic steatosis and a more significant percent weight loss (7-10%) to improve features of NASH [17]. Although there is a lack of global consensus on a specific dietary strategy, the Mediterranean diet, rich in omega-3-unsaturated polyunsaturated fatty acids, is recommended by multiple European organizations as the best solution [148].

Pharmacological intervention is indicated only in the case of NASH [17]. The use of several pharmacologic agents has been explored; however, the supporting data are limited and largely empirical with many of their clinical trials occurring in uncontrolled environments. Currently, pioglitazone, a thiazolidinedione, is the only insulin-sensitizing agent recommended by AASLD to patients with NASH [17]. Vitamin E, with its antioxidant anti-inflammatory and anti-apoptotic properties, is recommended by AASLD only in nondiabetic patients with biopsy-proven NASH [17]. Monitoring of older male patients is indicated during continuous use for more than three years, due to an increased risk of prostate cancer [149-151]. Lipid-lowering agents (statins, fibrates, and ezetimibe) can reduce FFAs and triglyceride deposition in hepatocytes, decreasing disease progression [152]. This exploits the positive correlation between subclinical atherosclerosis and progression and mortality in NASH patients [152]. Surgical management of NASH is performed in cases of complications such as cirrhosis or HCC [153]. NASH-related cirrhosis and HCC are increasingly common indications for liver transplantation in the West [154]. Bariatric surgery has been shown to reverse histological features of NASH; however, it is not recognized as a treatment for NASH and is only indicated in severe obesity to aid weight loss [17,155].

The use of obeticholic acid has been found to noticeably improve the histological changes in patients with early stages of fibrosis in NASH, but the outcome is unsatisfactory in advanced NAFLD. In individuals with T2DM and NAFLD, the use of sodium-glucose cotransporter-2 (SGLT-2) inhibitors, like empagliflozin and dapagliflozin, lowers liver fat and raises ALT levels [153,154]. Newer thiazolidinediones like saroglitazar (a dual peroxisome proliferator-activated receptor (PPAR) α/γ agonist, approved for diabetic dyslipidemia) and elafibranor (an agonist of PPAR-α and PPAR-δ) have shown improvement in insulin resistance and serum lipid normalization, which are two important causes of NASH progression. The safety profile is excellent, although it causes reversible serum creatinine increases, which may limit its use in individuals with a renal illness. It calls for the personalization of drug combinations, depending on the stage of NASH and associated morbidities for each patient [154].

Established medications are being explored as potential therapies for NAFLD. For example, liraglutide, a glucagon-like peptide 1, has been demonstrated to cause a resolution in the histological features of NASH and can be considered as a potential treatment [156]. Other naturally occurring substances such as silymarin, betaine, quercetin, and probiotics have shown mixed results and may be used along with other therapies [157]. Several new medications targeting pathways driving progression are also in development. Cenicriviroc, a chemokine antagonist targeting pro-inflammatory pathways, was investigated for NASH treatment [158]. Selonsertib, which modulates cell death via apoptosis signal-regulating kinase-1, was considered [159]. Drugs targeting fibrogenesis such as simtuzumab were also researched [160]. However, their viability as treatments remains limited, possibly due to the complexity of the targeted pathways. Development of new pharmacological agents and their validation through large-scale randomized controlled trials are required to offset the increase in NASH-indicated liver transplantations.

## Conclusions

NAFLD is a very broad disease, with multiple risk factors acting in concert to manifest its numerous clinical consequences. The incidence of risk factors associated with this disease, such as diabetes mellitus, for instance, is increasing rapidly across the world, increasing the prevalence of NAFLD. Despite repeated efforts by multiple teams from across the world, there is still no cure for NAFLD. Therefore, its management is focused on the prevention and early detection of this disease.
